# Antimicrobial Resistance in the Netherlands: A Natural Experiment?

**DOI:** 10.3389/fpubh.2014.00005

**Published:** 2014-01-24

**Authors:** Christina Maria Joseph Elisabeth Vandenbroucke-Grauls

**Affiliations:** ^1^Department of Medical Microbiology and Infection Control, VU University Medical Center, Amsterdam, Netherlands

**Keywords:** antimicrobial resistance, resistant strains, human antibiotic use, veterinary antibiotic use, travel

Use and misuse of antimicrobial agents lead to antimicrobial resistance. This is well-known, as it is equally well known that restrictive use results in lower resistance rates. However, how powerful restrictive use is in minimizing the rise of antimicrobial resistance is not very well documented.

There are examples in the literature of changes in antibiotic use that have led to lowering resistance rates. Well-known is the drop in erythromycin resistance in Finland after a policy change with respect to outpatient treatment of respiratory and skin infections (1). In Iceland, Kristinsson et al. showed the relation between antimicrobial use and the epidemiology of resistant pneumococci (2). Also in the hospital setting, lowering the use of specific antibiotics impacts resistance rates (3, 4). Still, examples of the positive effect of reduction of antibiotic use on resistance rates are scarce, and reasonable doubt exists about the possibilities to reverse resistance once it is established. Antibiotic resistance comes at a fitness cost, and has an effect on bacterial growth rate. This should lead to outgrowth of the susceptible strains of bacteria when the selective pressure of antibiotics is reduced. Andersson and Hughes reviewed the available data and conclude that the rate of reversibility is probably very low, due to the acquisition of mutations that compensate for the loss of fitness (5).

Less explored is the effect of low vs. high antibiotic use on the introduction and transmission of resistant strains. Possibly, antibiotic use may affect the ease and rapidity by which resistant strains are transmitted and establish themselves in new hosts. If this is true, it would mean that low antibiotic use in a population can help to resist the introduction and spread of resistant strains in that population, even when the pressure for introduction is high. The Netherlands (and other countries with low antibiotic use) may serve as a model for this assumption. The Netherlands has the lowest human antibiotic consumption rate of Europe: this was 11.4 defined daily doses (DDD) per 1000 inhabitants/day in 2011; high antibiotic-consuming countries used three times as much (32.0 and 35.1 DDD per 1000 inhabitants/day in Cyprus and Greece, respectively) (6). At the same time, this country belongs to the large consumers of veterinary antibacterial agents in Europe: between 2005 and 2009 it had among the highest sales of antibiotics for veterinary use of 10 European countries investigated by Grave et al. (7, 8). In particular, sales of third- and fourth-generation veterinary cephalosporins were high in The Netherlands (8). The effect of this use was, among others, reflected in high resistance rates to cephalosporins in *E. coli* in broiler chickens (9), and resulted in high contamination rates of chicken meat with extended-spectrum beta-lactamases (ESBL)-producing enterobacteriaceae (10). Contamination with ESBL-producing strains was found in up to 90% of retail chicken meat samples (10, 11). Another veterinary indicator of high antibiotic use is the massive colonization of Dutch pigs with methicillin-resistant *Staphylococcus aureus* (MRSA) (12). Resistant strains are also found in the Dutch soil (13, 14) and on vegetable produce (15, Reuland et al., submitted for publication).

Taken together, this means that the Dutch human population lives in an ecosystem that is brimming with antibiotic resistant strains, with the large use of antibiotics in livestock farming. In addition, resistant strains are continuously introduced by travelers. Yearly, nearly 15 million outbound holiday trips are booked by the Dutch; of these, approximately 3 million are spent outside Europe (16). These figures do not include business travel. Initially, it was thought that resistant strains are brought to Dutch hospitals mainly by patients who are repatriated after a stay in foreign hospitals: a study performed in 2004 showed that nearly one in five patients coming from foreign hospitals is colonized with resistant bacteria (17). Now, 10 years later, several studies have shown that travel in itself, without hospitalization in the foreign country is a risk factor for acquisition of resistant strains. These recent studies have focused on bacteria producing ESBL and carbapenemases. The first study was a Swedish study that addressed colonization of Swedish travelers and showed that 24% of healthy persons who were not colonized with ESBL-producing strains prior to travel, were colonized upon return (18). A second study was performed in the Netherlands by Paltansing and colleagues (19). These authors showed that a little less than one third of travelers become colonized with ESBL-positive bacteria during travel. Half of these still carried resistant strains after 6 months.

Despite the constant presence of resistant strains in their surrounding and the continuous introduction of resistant strains through travel, resistance in humans in The Netherlands is low when compared to other European countries. This is shown year after year by the surveillance data reported annually by EARS-NET (European Antimicrobial Resistance Surveillance System of ECDC) (20). Thus, low antibiotic use in a population seems to minimize the spread of antimicrobial resistance, even if this population is embedded in an antibiotic-rich environment and continuously threatened by introduction of new resistant strains.

Still, it should be noted that these low rates of resistance are slowly increasing, and the rise is a cause of concern. It is as yet impossible to estimate what the main contributors are. As a result of widespread concern, a movement toward lower antibiotic use in animal husbandry has been started. The latest report on monitoring of antimicrobial resistance and antibiotic usage in animals in the Netherlands (21) shows that total sales of antibiotics for veterinary use have dropped by approximately 50% between 2007 and 2012. Among others, the use of third generation cephalosporins was completely stopped in March 2010 in broilers and in pigs. This is accompanied by a reduction in resistance in colonizing *E. coli* in chicken, pigs, and calves. The future will tell whether this change is sufficient to slow down the rising resistance in humans.
